# Experimental effects of exposure to media images in adolescent girls: role of appearance comparison and peer norms for appearance

**DOI:** 10.1186/2050-2974-2-S1-O42

**Published:** 2014-11-24

**Authors:** Siân A McLean, Susan J Paxton, Eleanor H Wertheim

**Affiliations:** 1 School of Psychological Science, La Trobe University, Melbourne, Australia

## 

Research has established that exposure to thin-ideal media images has a small negative effect on body image. Less is known about the processes that mediate this effect. In an experimental design, this study aimed to compare the effect on body satisfaction of exposure to thin-ideal images in three conditions: 1) Control, 2) Appearance Comparison (AC), and 3) Peer Appearance Norms (PN). Participants were 140 female year 7 students who viewed 10 media images, before and after which they completed measures of body satisfaction. During exposure, participants responded to questions about the images which focused on design features for control, comparison of oneself with the images for AC, or consideration of peers' appearance-based judgements of others for PN participants. Analysis of covariance revealed a significant effect of condition on body satisfaction. Post-hoc tests indicated that the AC group had significantly lower post-exposure body satisfaction than both control and PN conditions. The latter two groups were not significantly different from one another. The results of this study add to and extend the small but growing body of evidence that engaging in appearance comparisons, but not consideration of peer appearance norms, while viewing images is a critical process in the manifestation of negative effects for body satisfaction.

This abstract was presented in the **Prevention & Public Health** stream of the 2014 ANZAED Conference.

